# Clinical Presentation and Management of Tubulointerstitial Nephritis and Uveitis Syndrome: A Case Series

**DOI:** 10.7759/cureus.101107

**Published:** 2026-01-08

**Authors:** João Cunha, Rita Afonso, Roberto Calças Marques, Ana Cabrita, Ana Paula Silva

**Affiliations:** 1 Nephrology Department, Unidade Local de Saúde do Algarve - Hospital de Faro, Faro, PRT

**Keywords:** corticosteroids, inflammation, tinu syndrome, tubulointerstitial nephritis, tubulointerstitial nephritis and uveitis syndrome, uveitis

## Abstract

Tubulointerstitial nephritis and uveitis (TINU) syndrome is a rare systemic inflammatory condition characterized by oculorenal involvement. The pathophysiology is not fully understood but is thought to involve an immune-mediated response, potentially triggered by infections, medications, or genetic factors. TINU syndrome is often challenging to diagnose due to the asynchronous presentation of ocular and renal manifestations, requiring the exclusion of other systemic diseases. We present a four-case series of TINU syndrome, diagnosed between 2014 and 2024 at a tertiary center, highlighting the diversity in clinical presentations and outcomes, with the aim of bringing the authors’ experience in the diagnosis and management of this still underrecognized syndrome.

## Introduction

Tubulointerstitial nephritis and uveitis (TINU) syndrome is a clinical entity first described in 1975 [1], with an increasing number of cases reported in the 21st century, rising from approximately 133 cases worldwide in the early 2000s to nearly 600 cases two decades later [2,3]. TINU syndrome is a systemic inflammatory disease that affects both the renal tubules and interstitium, as well as the uveal tissue of the eye, often presenting asynchronously, with ocular manifestations ensuing the diagnosis of tubulointerstitial nephritis in 65% of cases. This characteristic makes the diagnosis of TINU syndrome particularly challenging [4,5].

Although the pathophysiology is not fully understood, it is believed to be an immune-mediated process involving both humoral and cellular responses [6]. There may also be a genetic susceptibility associated with various human leukocyte antigens (HLAs), and several triggers, including infections and drugs, appear to contribute to the onset of clinical presentation [7,8]. The differential diagnosis of TINU syndrome is extensive, as many diseases can lead to oculorenal involvement, including autoimmune diseases (e.g., sarcoidosis, Sjogren’s syndrome (SS), systemic lupus erythematosus (SLE)) and infectious diseases (e.g., tuberculosis, infectious mononucleosis, toxoplasmosis). For this reason, TINU syndrome is considered a diagnosis of exclusion [2].

In this report, we describe four clinical cases of TINU syndrome, each one with distinct clinical presentations, therapeutic approaches, and outcomes, which highlight the heterogeneity of this pathology, despite the relatively few cases documented in the literature.

## Case presentation

Case 1

A 68-year-old woman presented with a two-month history of reduced visual acuity and retro-orbital headache, initially affecting the left eye and later involving the right. The patient also reported unintentional weight loss (8 kg in two months), along with postprandial nausea and vomiting. A diagnosis of bilateral anterior uveitis was made, and topical therapy was prescribed for one month with topical corticosteroids and tropicamide 10 mg/mL (one drop in the morning), leading to improved ocular inflammation.

Initial laboratory workup highlighted a normocytic normochromic anemia (hemoglobin (Hb): 8.2 g/dL), an elevated erythrocyte sedimentation rate (ESR) (80 mm/hour) and serum creatinine (sCr) (4.4 mg/dL), mild proteinuria (24-hour urine protein: 369 mg), and sterile leukocyturia (25 white blood cells/high-power field). Renal ultrasound showed kidneys at the lower limit of normal size with slight loss of corticomedullary differentiation and no evidence of hydronephrosis. Further investigations, including viral serologies (hepatitis B, hepatitis C, and human immunodeficiency virus), autoimmune tests (antinuclear and antineutrophil cytoplasmatic antibodies and anti-glomerular basement membrane antibodies), angiotensin-converting enzyme, protein electrophoresis, and serum free light chains measurements, were normal, except for a positive lupus anticoagulant that was not confirmed upon repeat testing. The interferon gamma release assay (IGRA) was negative.

**Figure 1:**
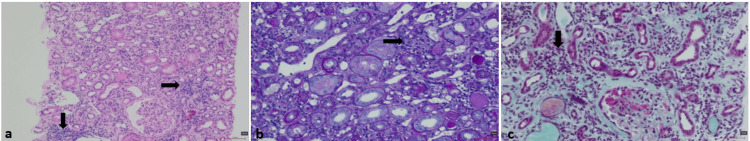
Kidney biopsy findings on light microscopy. Hematoxylin and eosin staining, original magnification ×250 (a), periodic acid-Schiff staining, original magnification ×400 (b), and Masson’s trichrome staining, original magnification ×400 (c), showing diffuse interstitial mononuclear infiltrate (arrows) with preserved glomerular and vascular compartments.

With a suspected diagnosis of TINU syndrome, treatment with intravenous isotonic saline and prednisolone (1 mg/kg/day; 65 mg/day) was initiated. A kidney biopsy confirmed the diagnosis, revealing a diffuse interstitial mononuclear infiltrate, preserved glomerular and vascular compartments, with no deposits on immunofluorescence (Figure 1).

Oral prednisolone was gradually tapered to 40 mg per day over three months, with plans for complete withdrawal over six months. However, the patient developed several adverse effects from systemic corticosteroids, including moon facies, buffalo hump, fluid retention, hypertension, and bone pain with confirmed fragility fractures of four dorsal vertebrae (without prior trauma). At this point, laboratory evaluation showed an sCr of 2.9 mg/dL and bland urinary sediment. Considering these findings, azathioprine (75 mg daily) was initiated, and prednisolone was tapered more rapidly. One month later, there was no significant improvement in kidney function, with an estimated glomerular filtration rate of 17 mL/minute/1.73 m² (sCr 2.8 mg/dL), according to the CKD-EPI 2021 equation, and a 24-hour urine protein of 219 mg. On the other hand, there was no further evidence of a relapse of ocular symptoms.

Case 2

A 49-year-old female presented with a one-month history of constitutional symptoms (unintentional weight loss of 6 kg, nausea, and vomiting) and polyuria. Concurrently, she developed ocular symptoms (one month before the onset of the constitutional symptoms), including ocular pain and blurred vision, first on the left, and then on the right eye, leading to a diagnosis of sequential bilateral anterior uveitis, which had good response to treatment with ocular drops of dexamethasone 1 mg/mL (one drop every two hours during the day) and cyclopentolate 10 mg/mL (one drop every eight hours during the day).

Initial laboratory workup revealed normocytic normochromic anemia (Hb: 10.8 g/dL), leukocytosis (leukocytes: 11,500/µL) with neutrophilia, elevated sCr (4.4 mg/dL), signs of tubular dysfunction that included mild hypokalemia (potassium: 3.2 mEq/L), hypophosphatemia (phosphorus: 2.1 mg/dL), hypouricemia (uric acid: 2.2 mg/dL), and glycosuria. Sterile leukocyturia (57 white blood cells/high-power field) and a non-nephrotic proteinuria (24-hour urine protein of 697 mg from 3,650 mL of urine) were found. Renal ultrasound was unremarkable. Extensive blood tests were negative for antinuclear antibodies, rheumatoid factor, and viral serologies (hepatitis B, hepatitis C, and human immunodeficiency virus), showing normal complement, angiotensin-converting enzyme, and serum free light chains, with no M protein detected on serum protein electrophoresis.

**Figure 2:**
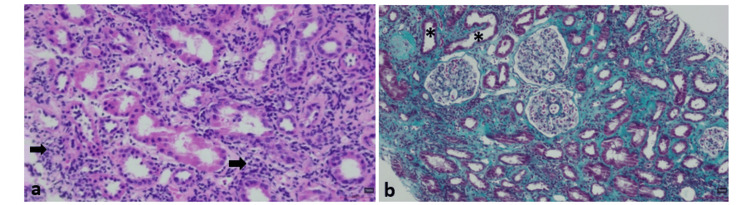
Kidney biopsy findings on light microscopy. Hematoxylin and eosin staining, original magnification ×400 (a) and Masson’s trichrome staining, original magnification ×250 (b), showing diffuse interstitial fibroedema with lymphocytic infiltrate (arrows), areas of tubulitis, and acute tubular necrosis (asterisks). Glomeruli are preserved.

Based on these findings, a diagnosis of TINU syndrome was presumed. Treatment with oral prednisolone was initiated at a dose of 1 mg/kg/day (65 mg/day), along with intravenous isotonic saline and management of electrolyte abnormalities. A subsequent kidney biopsy confirmed the diagnosis, revealing preserved glomeruli, diffuse interstitial fibroedema with lymphocytic infiltrate, areas of tubulitis, and acute tubular necrosis, with negative immunofluorescence (Figure 2).

One month after starting systemic corticosteroids, the patient showed significant improvement in kidney function, with an sCr level of 1.2 mg/dL and reduced leukocyturia (5 white blood cells/high-power field) and proteinuria (urine protein/creatinine ratio of 140 mg/g), despite some adverse effects, including moon facies and insomnia, prompting to a quicker tapering schedule of prednisolone. After four months of follow-up, and already without prednisolone, there was no evidence of renal or ocular relapse.

Case 3

A 27-year-old female with a history of seronegative rheumatoid arthritis and obesity, taking sulfasalazine, cyclobenzaprine, and as-needed etoricoxib, presented with a two-day history of anorexia, nausea, and hypogastric/iliac pain.

On admission, the patient had signs of dehydration, and the initial laboratory workup showed normocytic normochromic anemia (Hb: 9.9 g/dL), elevated C-reactive protein (CRP) (137 mg/L), ESR of 64 mm/hour, and elevated sCr (14.7 mg/dL). Urinalysis showed blood, protein, leukocytes, and nitrites. Renal ultrasound excluded signs of chronicity and hydronephrosis. A urinary tract infection was initially suspected, and the patient was started on amoxicillin-clavulanate and intravenous fluids. Given the potential for sulfasalazine and etoricoxib to cause acute kidney injury, particularly acute interstitial nephritis (AIN), both medications were discontinued. Microbiologic cultures of blood and urine were negative. Further immunologic, serologic, and urinary studies revealed polyclonal gammopathy, non-nephrotic proteinuria (24-hour urine protein: 1,016 mg, predominantly non-albumin), and sterile pyuria (6 white blood cells/high-power field). The patient’s symptoms resolved with continued treatment (intravenous fluids and antibiotic therapy for 14 days), and after three weeks, she was discharged with a significant reduction of sCr to 2.9 mg/dL.

**Figure 3:**
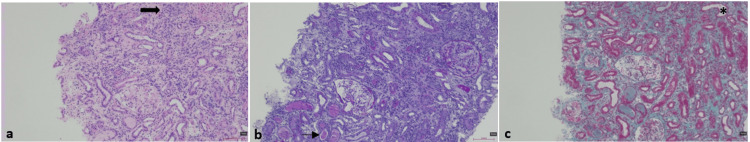
Kidney biopsy findings on light microscopy. Hematoxylin and eosin staining, original magnification ×250 (a), periodic acid-Schiff staining, original magnification ×250 (b), and Masson’s trichrome staining, original magnification ×250 (c), showing interstitial fibroedema, tubulointerstitial cellular infiltration (predominantly lymphocytes, large arrow), acute tubular necrosis (asterisk), and epithelial cell casts (narrow arrow). Glomeruli are preserved.

Two weeks later, the patient returned complaining of nausea and vomiting, with worsening kidney function (sCr: 3.7 mg/dL), mild hypokalemia (3.2 mEq/L), and isomorphic microscopic hematuria (5 red blood cells/ high-power field), on complementary examinations. This time, it was decided to perform a kidney biopsy, which showed normal glomeruli, interstitial fibroedema, tubulointerstitial cellular infiltration (predominantly lymphocytes), acute tubular necrosis, and epithelial cell casts, with no deposits on immunofluorescence (Figure 3).

A diagnosis of AIN of unknown etiology was made, with possible drug-induced or infection-related causes. Treatment with prednisolone at a dose of 1 mg/kg/day (80 mg/day) was initiated, and a significant improvement of renal function and proteinuria was seen (sCr: 1.5 mg/dL, 24-hour proteinuria: 600 mg), leading to a rapid taper of systemic corticosteroids (in approximately one month). However, a few days after the oral prednisolone course was finished, proteinuria and sCr increased to 1,026 mg/24 hours and 1.8 mg/dL, respectively, prompting a reintroduction of prednisolone (in a dose of 20 mg/day), followed by a slower tapering regimen.

Six months later, while tapering to 2.5 mg/day of prednisolone, the patient developed bilateral ocular symptoms, including burning, photophobia, redness, and decreased visual acuity. Ophthalmological examination confirmed bilateral anterior uveitis, which improved with topical steroids, without the need to increase systemic prednisolone. A final diagnosis of TINU syndrome was established. At 12 months, after completing the corticosteroid taper, proteinuria resolved (122 mg/24-hour), and renal function stabilized (sCr: 1.2 mg/dL) during the five years of follow-up that the patient already had.

Case 4

A 24-year-old obese male presented with a two-week history of nausea, vomiting, right flank pain, and watery diarrhea. During this period, he had been taking ibuprofen for flank pain relief.

Upon examination, no significant findings were noted. Laboratory workup showed normocytic normochromic anemia (Hb: 12.6 g/dL), leucocytosis (leucocytes: 13,300/µL) with neutrophilia, elevated ESR (102 mm/hour), elevated CRP (139 mg/L), and elevated sCr (3.5 mg/dL). Urinalysis revealed proteins (++) and blood (+), with 25 leucocytes/high-power field. Empiric antibiotic with amoxicillin-clavulanate and intravenous fluids were initiated for suspected infection (acute pyelonephritis vs. gastroenteritis) and prerenal acute kidney injury. Further evaluation, including urine culture, viral serologies (hepatitis B, hepatitis C, and human immunodeficiency virus), autoimmune tests (antinuclear and antineutrophil cytoplasmic antibodies and anti-glomerular basement membrane antibodies), angiotensin-converting enzyme levels, protein electrophoresis, and serum free light chains measurements, was negative. A 24-hour urine protein of 416 mg was noted. Despite modest clinical improvement and decreasing inflammatory markers, sCr continued to rise, reaching a maximum of 5.6 mg/dL after five days of hospital admission. AIN was suspected, potentially due to unidentified infection or ibuprofen use, and systemic corticosteroids were started (5 days of 500 mg of intravenous methylprednisolone, followed by 80 mg/day of oral prednisolone). The patient improved rapidly, with sCr decreasing to 2.6 mg/dL at discharge. One month later, sCr decreased to 1.2 mg/dL, and 24-hour urine protein dropped to 210 mg, allowing for tapering of prednisolone.

**Figure 4:**
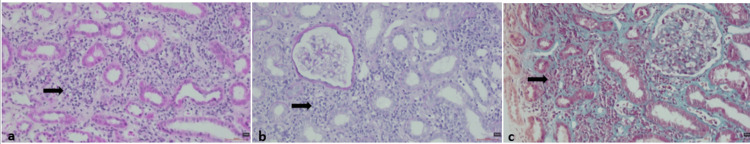
Kidney biopsy findings on light microscopy. Hematoxylin and eosin staining, original magnification ×400 (a), periodic acid-Schiff staining, original magnification ×400 (b), and Masson’s trichrome staining original magnification ×400 (c), showing interstitial edema and lymphocytic infiltration (arrows), with preserved glomeruli.

During the prednisolone tapering (at 12.5 mg/day), both sCr and 24-hour urine protein increased to 1.5 mg/dL and 355 mg, respectively, prompting a kidney biopsy and an increase in prednisolone dosage to 20 mg/day. The kidney biopsy confirmed AIN, with normal glomeruli, interstitial edema, and lymphocytic infiltration (Figure 4). Immunofluorescence was negative.

Five months after the kidney biopsy and 10 months after the onset of symptoms, with improvement of kidney function and proteinuria, and while on 15 mg/day of prednisolone, the patient developed pain, redness, photophobia, and decreased visual acuity in both eyes. Ophthalmologic examination confirmed non-granulomatous bilateral anterior uveitis. Topical steroid drops were added, and the oral prednisolone dose was maintained. A diagnosis of TINU syndrome was established based on these findings.

In the following months, the patient experienced several relapses of uveitis, but sCr remained stable (1.2 to 1.4 mg/dL), and proteinuria resolved. Nine months after the initial uveitis diagnosis, both ocular and systemic steroids were discontinued following the resolution of ocular symptoms.

Table 1 provides a summary of patient presentations of all four cases.

**Table 1:** Highlights of clinical cases. AIN = acute interstitial nephritis; AKI = acute kidney injury; ATN = acute tubular necrosis; CKD = chronic kidney disease; CRP = C-reactive protein; ESR = erythrocyte sedimentation rate; Hb = hemoglobin; HPF = high-power field; IV MTP = intravenous methylprednisolone; KDIGO = Kidney Disease: Improving Global Outcomes; PCR = protein-creatinine ratio; RBC = red blood cells; WBC = white blood cells

Characteristics	Case 1	Case 2	Case 3	Case 4
Gender	Female	Female	Female	Male
Age at diagnosis (years)	68	49	27	24
Trigger(s)	None	None	None (suspected urinary tract infection)	None (suspected viral gastroenteritis)
Accompanying symptoms	Weight loss, nausea, and vomiting	Weight loss, nausea, vomiting, and polyuria	Anorexia, nausea, vomiting, and abdominal pain	Nausea, vomiting, abdominal pain, and watery diarrhea
Uveitis	Bilateral (additive); anterior	Bilateral (alternating); anterior	Bilateral (initially); anterior	Bilateral (initially); anterior
Uveitis onset	2 months before kidney abnormalities	2 months before kidney abnormalities	6 months after kidney abnormalities	10 months after kidney abnormalities
AKI stage (KDIGO)	KDIGO 3	KDIGO 3	KDIGO 3	KDIGO 3
Hb (g/dL) at admission (normal range: 11.5–15.5 for females; 13.5–17.5 for males)	8.2	10.8	9.9	12.6
Leucocyte count (/µL) (normal range: 4,500–11,000)	6,000	11,500	9,100	13,300
ESR (mm/hour) (normal range for ≤50 years old: <20 for females; <15 for males. Normal range for >50 years old: >30 for females; >20 for males)	80	-	64	102
CRP (mg/L) (normal range: <10 mg/L)	<1	9	137	139
Electrolyte abnormalities	Hyperchloremic metabolic acidosis	High anion gap metabolic acidosis; hypokalemia; hypophosphatemia; hypouricemia	High anion gap metabolic acidosis; hypokalemia	None
Urinary abnormalities	Proteinuria (+); leukocyturia (25 WBC/HPF); uroepithelial cells; granular casts	Proteinuria (+); glycosuria (+++); leukocyturia (57 WBC/HPF); renal epithelial cells	Proteinuria (++); nitrituria (+); leukocyturia (6 WBC/HPF)	Proteinuria (++); Leukocyturia (25 WBC/HPF); non-dismorphic erythrocyturia (5 RBC/HPF)
PCR (mg/g) (normal range: <150)	585	658	1,198	263
24-hour urinary proteins (mg) (normal range: <150)	369	697	1,016	419
Autoimmunity/Serologic findings	Positive result for lupus anticoagulant (not confirmed)	Negative	Polyclonal gammopathy	Negative
Kidney biopsy findings	AIN, ATN, arteriosclerosis	AIN, ATN	AIN, ATN	AIN
Ocular treatment	Steroid, and anticholinergic ocular drops	Steroid, and anticholinergic ocular drops	Steroid ocular drops	Steroid ocular drops
Systemic treatment				
First-line therapy	Oral prednisolone at 65 mg/day, with slow tapering	Oral prednisolone at 65mg/day, with slow tapering	Oral prednisolone at 80 mg/day, with quick tapering	5 days of IV MTP pulses (500 mg), followed by oral prednisolone at 80 mg/day, with slow tapering
Second-line therapy	Azathioprine 75 mg/day		Oral prednisolone at 20 mg/day, with slow tapering	Oral prednisolone at 20 mg/day, with slow tapering
Follow-up (months)	8	4	60	132
Ocular relapse	No	No	Yes	Yes
Renal relapse	Progression to CKD, stage G4A2	No	No	No

## Discussion

Epidemiology

Published data on TINU syndrome estimate an incidence of 1-2 cases per 10 million individuals per year and a prevalence of 3.5 cases per million, corresponding to 5% of tubulointerstitial nephritis biopsies, and to 0.1-2% of uveitis cases [7-9]. However, the true prevalence may be higher than currently recognized. In our hospital, two of the four cases were identified in the same year, indicating that this is a syndrome for which we should maintain a high degree of suspicion when evaluating patients with kidney injury of uncertain etiology. TINU syndrome exhibits a female predominance (female-to-male ratio of 1.8) and has been reported across all age groups, with a median age of onset of 17 years, according to the most recent systematic review published in 2022 [2]. In our series, three patients were female (75%), and the mean age at diagnosis was 42 years (range = 24-68 years), which is older than previously reported in the literature. This shift in epidemiology is likely due to increased awareness of the disease in recent years.

Pathophysiology

The exact pathophysiology of TINU syndrome remains unknown; however, several potential triggers have been proposed, including genetic and environmental factors [8,10-12]. Medications such as non-steroidal anti-inflammatory drugs (NSAIDs), antibiotics, and prior infections (e.g., Epstein-Barr virus, herpes zoster virus, and *Chlamydia trachomatis*) have been suggested as potential contributors [2,13,14]. Additionally, autoimmune diseases such as rheumatoid arthritis and hyperthyroidism have been proposed as possible associations, although it remains uncertain whether these links are coincidental or causative. Some of these potential triggers were identified in our cases. One patient had a medical history of seronegative rheumatoid arthritis, used etoricoxib as needed, and had a urinary tract infection. Another patient was presumably diagnosed with an infectious gastroenteritis. Genetic susceptibility has also been reported, with associations to various HLA phenotypes. Levinson et al. identified the HLA-DQA1*01/DQB1*05/DRB1*01 haplotype in 13 of 18 patients with TINU syndrome, suggesting that these HLA variants may be risk factors for the development of the disease [15].

Clinical features

There is a wide spectrum of clinical presentations in TINU syndrome. Systemic symptoms are common, with fatigue, weight loss, and fever being the most frequently reported complaints [2]. In our series, all patients presented with nausea and vomiting, and two patients experienced abdominal pain and weight loss. One patient also reported anorexia. Renal manifestations included tubular low-grade proteinuria (n = 4), with three patients having a 24-hour urine protein under 1,000 mg, sterile pyuria (n = 4), and hematuria (n = 1). Additionally, all patients presented with stage 3 acute kidney injury, with a mean sCr at the time of presentation of 4 mg/dL (range = 3.5-4.4 mg/dL). Tubular defects may also occur, but complete proximal tubular abnormalities have rarely been described [1,8]. In Case 2, the patient exhibited some characteristics suggestive of proximal tubular defects, including glycosuria, polyuria, hypokalemia, hypouricemia, and hypophosphatemia. Despite not being measured in the reported cases, there has been a growing awareness of the role of urinary biomarkers, namely, beta-2 microglobulin. In TINU syndrome, this protein can be elevated in the urine even when sCr is in the normal range, showing some correlation with the histopathological grade of AIN. However, urinary beta-2 microglobulin can be elevated in any cause of AIN, making it a non-specific biomarker of TINU syndrome [16,17]. Normocytic normochromic anemia and an elevated ESR were present in all cases, except for one patient in whom the exact value of the elevated ESR was unknown.

Uveitis was anterior and bilateral in all cases, consistent with the literature, which reports 65% and 88% of cases as anterior and bilateral, respectively [2]. Ocular symptoms included reduced visual acuity (n = 3), ocular pain/retro-orbital headache (n = 3), photophobia (n = 2), redness (n = 2), blurred vision (n = 1), and burning (n = 1). Ocular symptoms either preceded or occurred after the renal diagnosis in equal proportions, with 50% of patients experiencing each pattern. In the latter scenario, the average delay in the development of uveitis was eight months; however, delays of up to 14 months have been reported in the literature, with a median onset of ocular symptoms of one month after the onset of systemic/renal symptoms [2,10]. This temporal discordance between clinical manifestations may lead to underdiagnosis and the misleading designation of “idiopathic” AIN. TINU syndrome should therefore be suspected in cases of “idiopathic” AIN in patients with a history of sudden-onset uveitis or who develop it after the diagnosis of this kidney disease.

Given that other systemic diseases can involve both the eyes and kidneys, TINU syndrome is a diagnosis of exclusion. Therefore, it is essential to conduct a thorough investigation, including autoimmunity testing. The differential diagnosis includes SS, sarcoidosis, SLE, Behçet’s disease, and infectious diseases such as toxoplasmosis and tuberculosis. Sarcoidosis is one of the most described diseases that shares clinical features with TINU syndrome, but it typically has pulmonary involvement, and kidney disease is mainly seen with concurrent hypercalcemia. Frequently, in this pathology, uveitis is granulomatous, and angiotensin-converting enzyme levels are elevated. Autoimmune diseases, such as SS and SLE, commonly manifest with ocular and kidney involvement, but while in SS, uveitis is rare, and patients have mainly dry eye, in SLE, kidney biopsies show almost always glomerular disease. Besides that, high titers of antinuclear antibodies support this group of diseases. Infectious diseases should not be forgotten in the differential diagnosis of TINU syndrome, with toxoplasmosis presenting with constitutional symptoms and posterior uveitis as ocular involvement, but primarily affecting immunocompromised hosts [2,16,18].

Typical histological renal findings include active interstitial inflammation primarily composed of lymphocytes, with fewer plasma cells and macrophages. Eosinophils and neutrophils can also be found in 34% and 25% of kidney biopsies, respectively. Glomeruli and vessels are generally preserved. No immune complexes are detected in immunofluorescence [9].

Treatment

Both topical and oral steroids (prednisolone 1 mg/kg/day) are used as first-line therapies in the TINU population to manage eye symptoms and treat AIN, respectively, despite the potential for spontaneous remissions. However, the evidence supporting this approach remains limited [2,13,19]. No prospective, randomized trials have compared steroid therapy with placebo or evaluated the optimal dose and duration of treatment. The tapering regimen is determined based on the clinician’s judgment and the patient’s clinical response. Other immunosuppressive agents, including methotrexate, mycophenolate, cyclosporine, azathioprine, and cyclophosphamide, have been used as second-line therapy [3]. Regarding uveitis, there is also evidence for the use of tumor necrosis factor-alpha inhibitors, such as adalimumab, particularly in patients who do not tolerate or fail to respond to oral steroids [20]. In our case series, one patient required a switch to azathioprine 75 mg/day due to the development of several serious adverse effects associated with systemic corticosteroids. Although systemic corticosteroids appear effective in controlling the disease, their toxicity cannot be overlooked, particularly in elderly individuals who are more susceptible. In this patient, one month after starting azathioprine, there was no improvement in renal function. Prospective multicenter trials are needed to determine the potential benefits of these and other therapeutic agents in TINU syndrome and to establish an optimal, stepwise therapeutic approach. While the overall kidney prognosis is generally favorable, incomplete recovery and progression to chronic kidney disease or end-stage renal disease have been observed in 2% of cases, underscoring the importance of controlled clinical trials to better identify patients at higher risk of unfavorable outcomes [9]. On the other hand, management of ocular involvement can be more challenging as uveitis may persist or relapse even after 10 years [2]. The long-term course of ocular and renal involvement appears to be independent. In our case series, patients who experienced ocular relapse did not show any worsening of kidney function, and the patient who did not show improvement in kidney function did not experience any ocular relapse.

## Conclusions

The incidence of TINU syndrome may be increasing due to heightened awareness of the disease. The diagnosis should be considered in the presence of nonspecific symptoms, with the understanding that there may be temporal dissociation between ocular symptoms and renal involvement. As such, a high degree of suspicion is crucial. TINU syndrome should be considered in patients with sudden-onset uveitis or idiopathic AIN. Further randomized, prospective, multicenter studies are warranted to standardize treatment protocols, evaluate the efficacy of second-line therapies, and more precisely identify patients at higher risk of adverse outcomes. Close collaboration between ophthalmologists and nephrologists is vital for ensuring appropriate follow-up care.
